# The Patterns of Coevolution in Clade B HIV Envelope's N-Glycosylation Sites

**DOI:** 10.1371/journal.pone.0128664

**Published:** 2015-06-25

**Authors:** Swetha Garimalla, Thomas Kieber-Emmons, Anastas D. Pashov

**Affiliations:** 1 University of Michigan Health System, Ann Arbor, MI, United States of America; 2 Winthrop P. Rockefeller Cancer Institute, University of Arkansas for Medical Sciences, Little Rock, AR, United States of America; 3 Stephan Angeloff Institute of Microbiology, Bulgarian Academy of Sciences, Sofia, Bulgaria; 4 UMRS872(equip 16), INSERM UMRS 872 Centre de Recherche des Cordeliers, Paris, France; University of California, San Francisco, UNITED STATES

## Abstract

The co-evolution of the potential N-glycosylation sites of HIV Clade B gp120 was mapped onto the coevolution network of the protein structure using mean field direct coupling analysis (mfDCA). This was possible for 327 positions with suitable entropy and gap content. Indications of pressure to preserve the evolving glycan shield are seen as well as strong dependencies between the majority of the potential N-glycosylation sites and the rest of the structure. These findings indicate that although mainly an adaptation against antibody neutralization, the evolving glycan shield is structurally related to the core polypeptide, which, thus, is also under pressure to reflect the changes in the N-glycosylation. The map we propose fills the gap in previous attempts to tease out sequon evolution by providing a more general molecular context. Thus, it will help design strategies guiding HIV gp120 evolution in a rational way.

## Introduction

Coevolving positions in the amino acid sequences of proteins are studied most often in terms of contact sites between or within protein molecules. Coevolution is considered also in terms of epistasis when functionally interdependent sites are involved [1, 2]. Further, distant sites could be bound by allosteric tracts [3, 4] and/or electrostatic interactions. The availability of large databases of homologous sequences and of methods refined for the detection of direct interactions (direct coupling analysis [5, 6]) are prerequisites for an increasingly reliable statistical analysis of coevolution.

A sizable collection of sequences is available from the genome of the highly variable HIV (http://www.hiv.lanl.gov/). Despite of a high rate of mutation the envelop proteinretains a dense glycosylation covering most of the exposed surface of the spike trimer. This shield consists of a relatively constant number of about 25 glycans per gp120 molecule [7] but almost all of the corresponding N-glycosylation sites are highly variable hence—“evolving glycan shield” [8].

The evolving glycan shield is an evolutionary concept implying complex interactions between N-glycosylation sites as part of an extensive network of structural interdependencies [9]. A major reason for the existence of such a dynamic structure is the necessity to shield neutralization epitopes on the protein surface within some structural constraints, e.g.—occlusion of the receptor and co-receptor binding sites, protomer interactions, molecular motions, etc. The immunological pressure is an evident but not isolated factor guiding the constant change of the sequon set. To the best of our knowledge, the only comprehensive study of the coevolution of the numerous gp120 N glycosylation sequons is the one by Wei, et al. [10]. The authors use a mutual information measure of coevolution with a level of significance determined by a bootstrap procedure, which is not sufficient to offset the entropy dependence and does not remove the chain effects. Consequently, the results are highly restricted and fail to demonstrate the obvious coevolution couplings for instance of adjacent and contacting residues.

Most studies of the structural aspects of Env gp120 to date have focused on clade B ([11–16] just to name a few). Utilizing this information, we studied the network of coevolving primary structure positions in Clade B gp120 based on a set of sequences from the database maintained at the Los Alamos National Laboratory (LANL) using mean field direct coupling analysis (mfDCA) [17]. The results were mapped onto the common residues involved in the structure 2B4C, which is also based on a Clade B isolate and features the constant core with the V3 loop in receptor and X5 antibody bound conformation. Previous studies using a different approach confirmed the hypothesis that immune pressure should lead to strong inter sequon dependencies [18] but failed to map inter sequon co-evolution onto the overall network of dependencies. Not unexpectedly, we found N-glycosylation sites to be engaged in numerous couplings within their network but also with other positions. Four positions often participating in sequons (E293, D322, S364 and N396) were found to be dependent predominantly on other sequons while positions N130, S199, N234, N241 and N362 were predominantly coupled to non-sequon positions. The rest of the sequons were dependent both on other sequon and non-sequon positions indicating that the immune pressure on the evolving glycan shield has a major impact on the structural stability of the gp120.

## Methods

### Sequences and models

To reduce the phylogenetic and sequence bias artefacts, a multiple sequence alignment was used that: 1) was restricted to one clade (B), 2) to one sequence per patient and 3) to sequences with less than 80% homology to any other. Ensuring a relatively homogenous evolutionary distance between the sequences avoids the phylogenetic artefact caused by deep trees with few main branches. This MSA (n = 984) is documented in S1 Dataset. In addition, positions were retained if they were less than 99% conserved and contained less than 20% gaps—this MSA is also part of S1 and S2 Datasets. The study was based on the clade B 2B4C crystal structure, which presents also the V3 loop but has a truncated V1/V2 loop and the N terminal 53 amino acids [13]. The final alignment contained 742 sequences and 327 positions with 64 sequons and 94 sequon related positions (SRP: P1 –N or P3 –S/T), of which respectively 40 and 61 are represented in 2B4C. The MSA was performed on the LANL server. The non-random occurrence of sequons was determined based on the hypothesis of random co-occurrence of N and S or T and the respective marginal frequencies in the MSA yielding 16 instance for this set of sequences. Thus, only those sequons were considered, which reoccurred 17 or more times at a particular position. Molecular graphics were generated using the UCSF Chimera package. Chimera is developed by the Resource for Biocomputing, Visualization, and Informatics at the University of California, San Francisco (supported by NIGMS P41-GM103311) [19].

### Mean field direct coupling analysis (mfDCA)

The method of mfDCA is described in detail elsewhere [5, 6, 17] and the algorithm in Matlab implementation is available at http://dca.upmc.fr/DCA/DCA.html. The essential property of this algorithm is that by using mean field approximation rather than belief propagation it provides a faster algorithm for eliminating the chain covariance effects and detects only the direct interaction between two positions in the sequence based on the observable covariances. Both methods can be viewed as “inference engines” acting on the statistical information encoded in large Bayesian networks [20]. The mfDCA results are found to outperform those generated by simple covariance analysis as well as approximate Bayesian analysis [17]. The mfDCA algorithm was applied to the Clade B MSA without change. The baseline mfDCA values were determined as the maximal mfDCA value for each position from 100 sequence alignments derived from the original MSA after scrambling separately each column. This preserves the marginal frequencies but destroys the inter position correlations (the script is in S2 File).

### Graph visualization

The set of relations was further analyzed as an undirected weighted graph and visualized using Gephi 0.8.2 software (www.gephi.org) [21]. For the graph analysis, the weight of the edges was calculated as:

$$w_{i,j}=-\text{log}D_{i,j},$$

where D_i,j_ is the mfDCA value of the significant couplings. Since 0<D<1, the lower weight corresponded to higher mfDCA value, which was necessary for the correct application of most of the graph analysis algorithms. The layout was generated by applying first a circular layout based on amino acid positions followed by ForceAtlas 2 algorithm (https://gephi.org/docs/toolkit/org/gephi/layout/plugin/forceAtlas/ForceAtlasLayout.html) with the following parameters: Dissuade Hubs, Prevent Overlap, Edge Weight Influence = 1.0, Scaling = 30, Gravity = 0.5, Tolerance Speed = 1.0–10.0 (changed twice between the extreme values). Finally the layout was Label Adjusted. The label sizes were scaled to the degree of the nodes. For the different presentations, the labels were color coded according to 1) their modularity class or 2) their sequon relation—first position (N), third position (S/T) or both when a variable site takes both values in different sequences. The gephi file containing the graph layout and the data table with the calculated graph parameters are available as supplement (S1 File).

### Betweenness centrality and all shortest paths

The betweenness centrality was calculated by the Ulrik Brandes algorithm as implemented in the Gephi suit [22] as well as by the David Gleich Matlab_BGL package based on the Boost graph Library (https://www.cs.purdue.edu/homes/dgleich/packages/matlab_bgl/) both algorithms giving the same result. The shortest paths were found using the Dijkstra algorithm as implemented in the GraphTheory Matlab package.

## Results

### Mean field direct coupling analysis of HIV gp120 Clade B sequences

**Fig 1:**
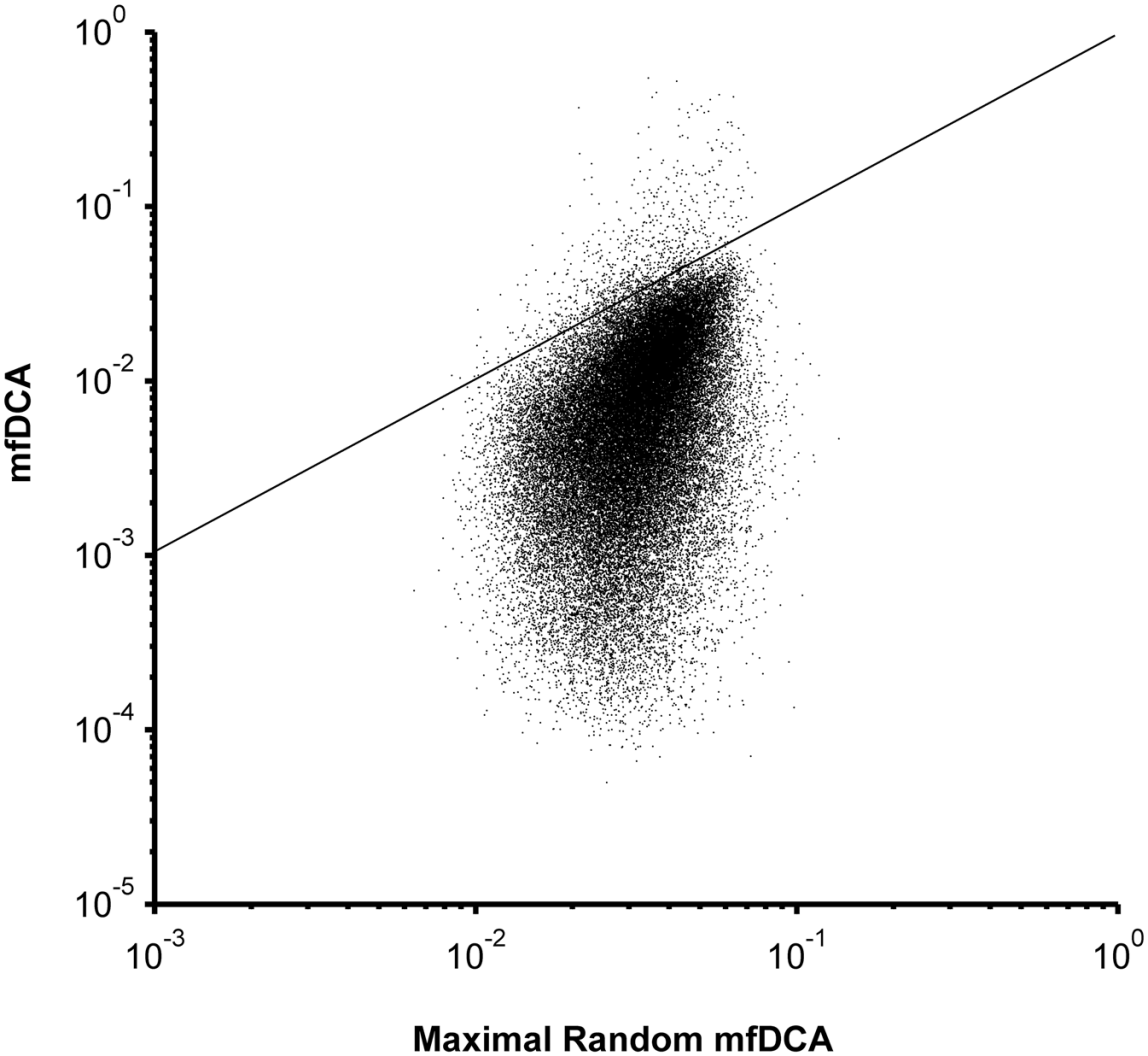
Comparison between mfDCA coupling values for Clade B gp120 based on the observed MSA or on the same MSA with scrambled columns. The amino acid residues in each column were randomly reordered and 100 different MSA with scrambled columns were thus generated. The mfDCA was performed on each of the 100 scrambled MSA and the maximal coupling values for each pair of positions (X) was related to the value from the observed MSA (Y). The maximum coupling value obtained after 100 permutations for each pair of positions was used as an upper limit of the random distribution of values for that pair. Thus, the dots above the diagonal represent the couplings, for which the actual mfDCA values were above the 99 percentile of the random coupling distribution. These mfDCA were considered significant and were used further in the analysis while the rest were replaced by zeros.

**Fig 2:**
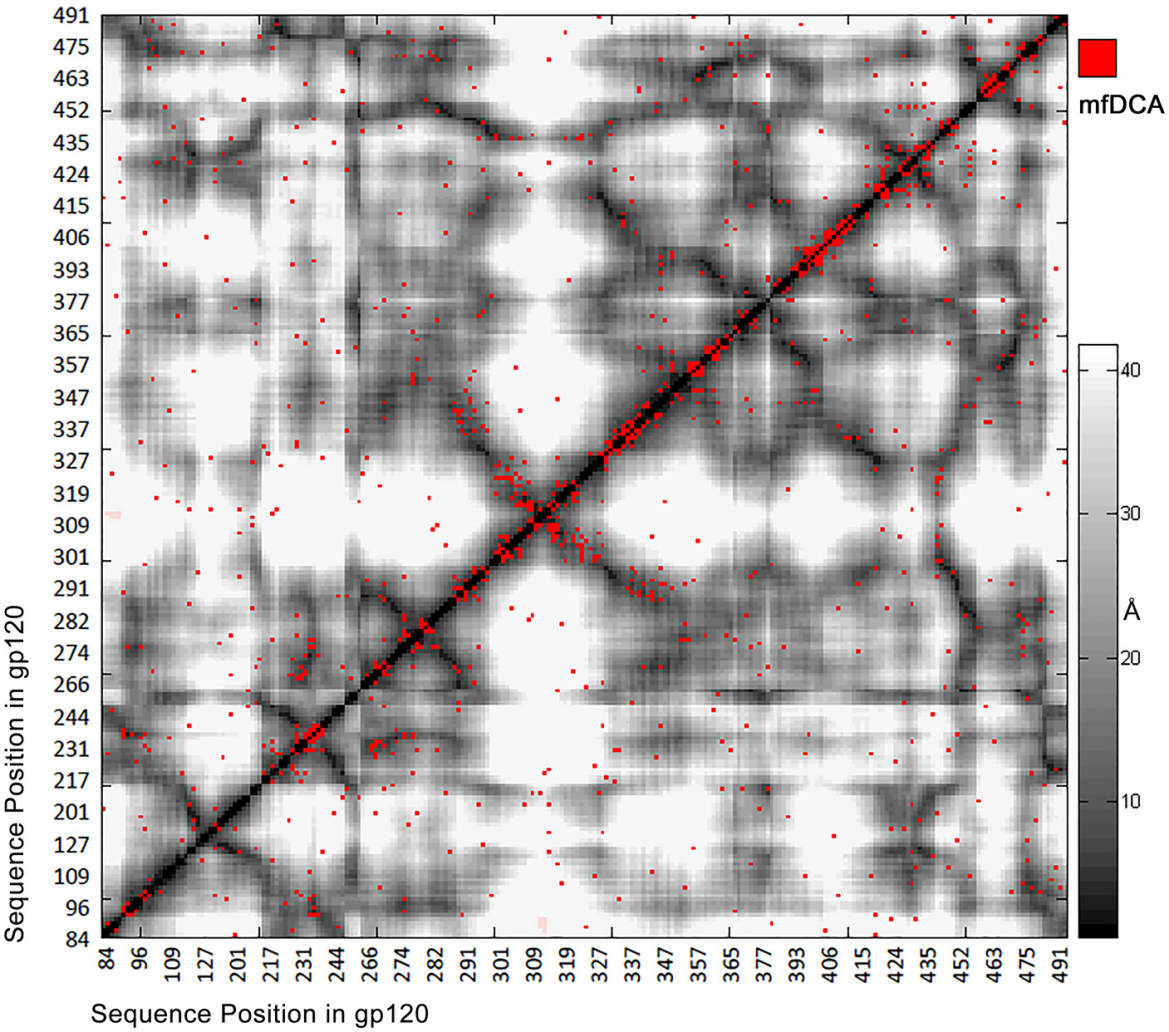
mfDCA coupling overlaid on the contact map of 2B4C.G structure. Significant couplings are marked with red dot to the background of gray level coded distances in angstroms. As expected, most of the significant values were along the diagonal indicating both primary and tertiary structure proximity. Those of highest intensity were aligned on the diagonal (not shown). A number of long range interactions (red dots in white areas) are observed also.

The network of coevolving positions was determined performing mfDCA on the multiple sequence alignment of 742 sequences with 327 considered positions. The mfDCA derived coupling values exceeding baseline (Fig 1) marked 969 significant of 53301 possible correlated pairs. The 2B4C structure contained 4356 pairs of positions with distances between the Cα atoms of less than 12 Å while the mfDCA algorithm highlighted 296 of those as functional couplings. As expected, the strongest interdependencies occurred for adjacent or nearby positions and also correlated inversely with the interatomic distances between the Cα atoms in the 2B4C structure (Fig 2 and S2 Fig). Nevertheless, 298 relations found by this analysis involved pairs at more than 12Å distance.

**Fig 3:**
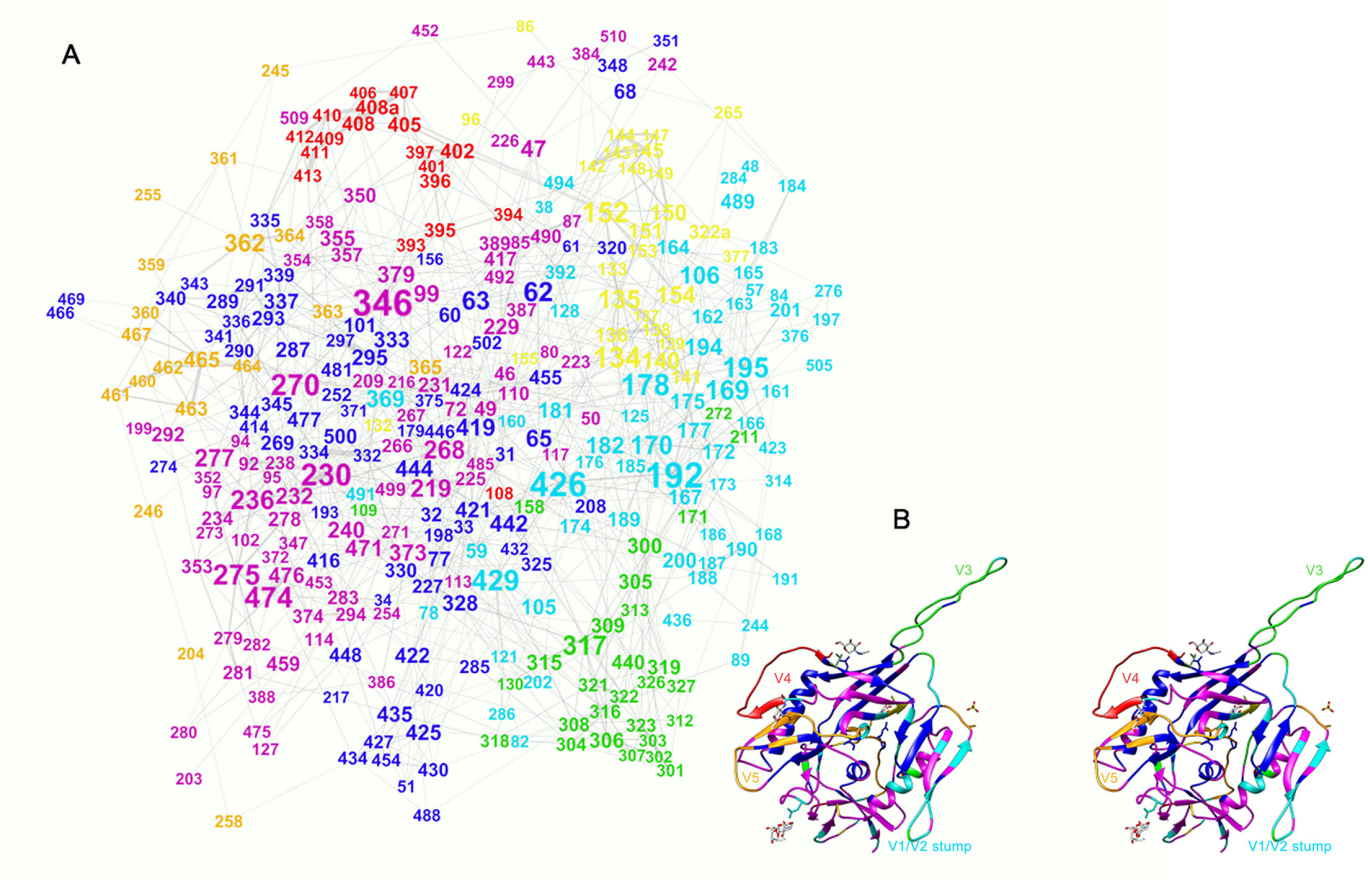
Graph representation of the interacting pairs. (A) The layout was constructed using Force Atlas 2 algorithm (Gephi). The size of the label (amino acid position number) correlates with the degree of the respective node in the graph. The modularity analysis of the graph yielded 7 modularity classes—color coded. The mapping of the major modularity classes on the 2B4C gp120 structure (B—stereo view) shows a good agreement with secondary and tertiary structure units. The V3 (green), V4 (red) and V5 (ochre) loops overlap with three separate classes, while V1/V2 fall into a class colored yellow. The logic of the visualization algorithm pulls cliques to the periphery and in the case of gp120 these are represented most notably by V4 and V5 and, to a lesser extent—V1/V2. Even the spatial relationships between these structures are marked although projecting on 2D plane distorts them. The only major discrepancy is the position of the V3 loop, but it is important to note that 2B4C structure represents the bound conformation of gp120 and the V3 loop in the unbound state seems to be positioned rather where the graph suggests. Thus the layout algorithm used reconstructed the main structural relations in gp120 based on the mfDCA coupling analysis.

The set of relations was further visualized as an undirected weighted graph with nodes representing the sequence positions and edges representing the significant couplings (Fig 3A). The graph consisted of a major component containing all analyzed positions except for 415 as an isolated node and 241 and 496, which formed the minor component. The mean degree was 5.92 meaning that on the average each position was coupled to approximately 6 others. The network diameter was 8. Thus, if the shortest paths between the aa positions in this direct couplings graph represent potential allosteric paths the longest among them is a chain of 8 residues.

### Visualization of the network of functional interactions

The visualization algorithm used—ForceAtlas2, is based on a classical force-directed layout with improvements allowing for better illustration of modularity [23]. At the same time a force-directed algorithm may be viewed as a very coarse simulation of molecular forces so the layout has the advantage of roughly representing a 2 dimensional projection of the structure. It only serves as an approximate layout, the spatial distribution of the nodes in which, resembling the actual protein structure, may serve as a check point for the relevance of the mfDCA data.

Indeed, a good agreement between the variable and constant domains of gp120 and the partition of residues in modularity classes of the graph was observed relating mostly to the capacity of mfDCA to predict contact residues and tertiary structure [17]. The densely connected positions in the variable loops formed cliques shown in the periphery of the graph (Fig 3). Interestingly, the apparent discrepancy between the position of V3 in the graph and the 2B4C structure can be explained by the bound conformation of the structure, while the coevolution network registered the strong relation of these residues to the outer domain—contacts V3 probably makes in the free conformation [24–26].

### Mapping the potential N-glycosylation sites

**Fig 4:**
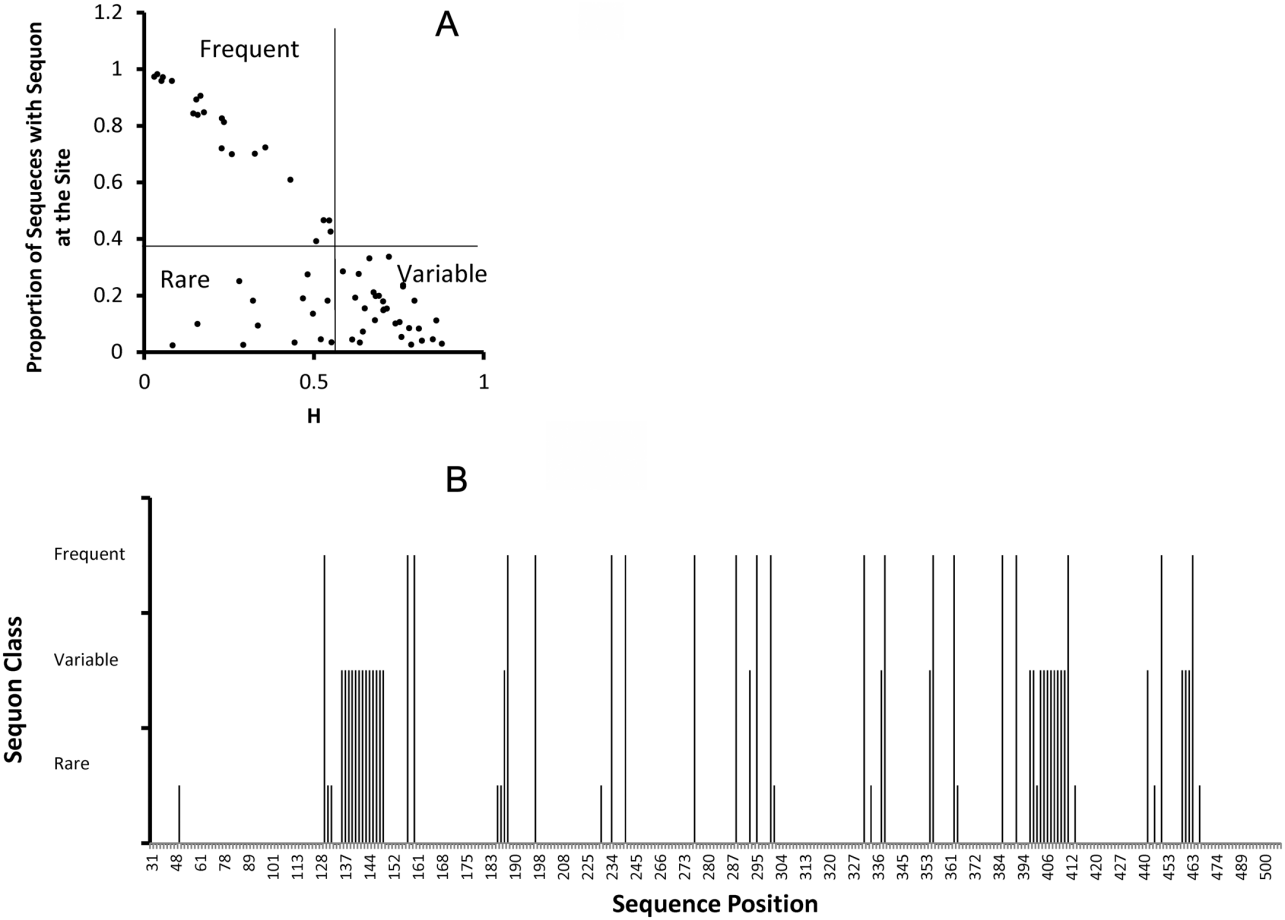
Distribution of potential N-glycosylation sites (sequons). Overall there were 94 positions, which represented less than 99% conserved sequon related asparagine residues. This excluded the two highly conserved positions N88 and N262. The lower bound was 16 sequences with sequon at the site—the threshold for random occurrence of sequons. As it has been demonstrated earlier, these sequons grouped further in several groups. Relating the frequency of the sequon in each of those positions to the entropy (variability) of the site (A), it was possible to outline three distinct groups. Some sites were represented more often than not by a sequon and were designated “Frequent”. Other, on the contrary, represented sequons in the minority of the sequences (although not random) and were designated “Rare”. The third group represented high entropy sites, in which neither the sequon related N nor other residues predominated—these were designated “Variable”. Mapping these three classes on the primary structure of gp120 (B) not surprisingly showed that “Variable” sequons were found in the variable regions, “Frequent” and “Rare” sequons appeared mostly on the constant and the “Rare” sequons were very often in close proximity to at least one sequon from the other classes.

Relating position entropy to frequency of sequon related asparagine grouped the potential N-glycosylation sites in 3 groups:—frequent (13 N and 10 S/T positions), rare (20 N and 14 S/T positions) and highly variable (31 N and 6 S/T positions) (Fig 4A). Some N positions coincide with S/T positions of an adjacent sequon so S/T positions are fewer. This classification omits the highly conserved positions N88 and N262, which were filtered initially. Since all the sequons represent only potential glycosylation sites from here on we shall omit the word potential but it will be implied all through this report. The complete list of the analyzed sequon positions is given in S1 Dataset. The variable positions are characteristic of the variable loops V1/V2, V4 and V5. The rare sequons occur actually in the close vicinity of frequent ones not only in the structure but also topologically in the graph. Therefore, most probably the rare sequons represent functionally equivalent variants of the frequent ones (Fig 4B).

**Fig 5:**
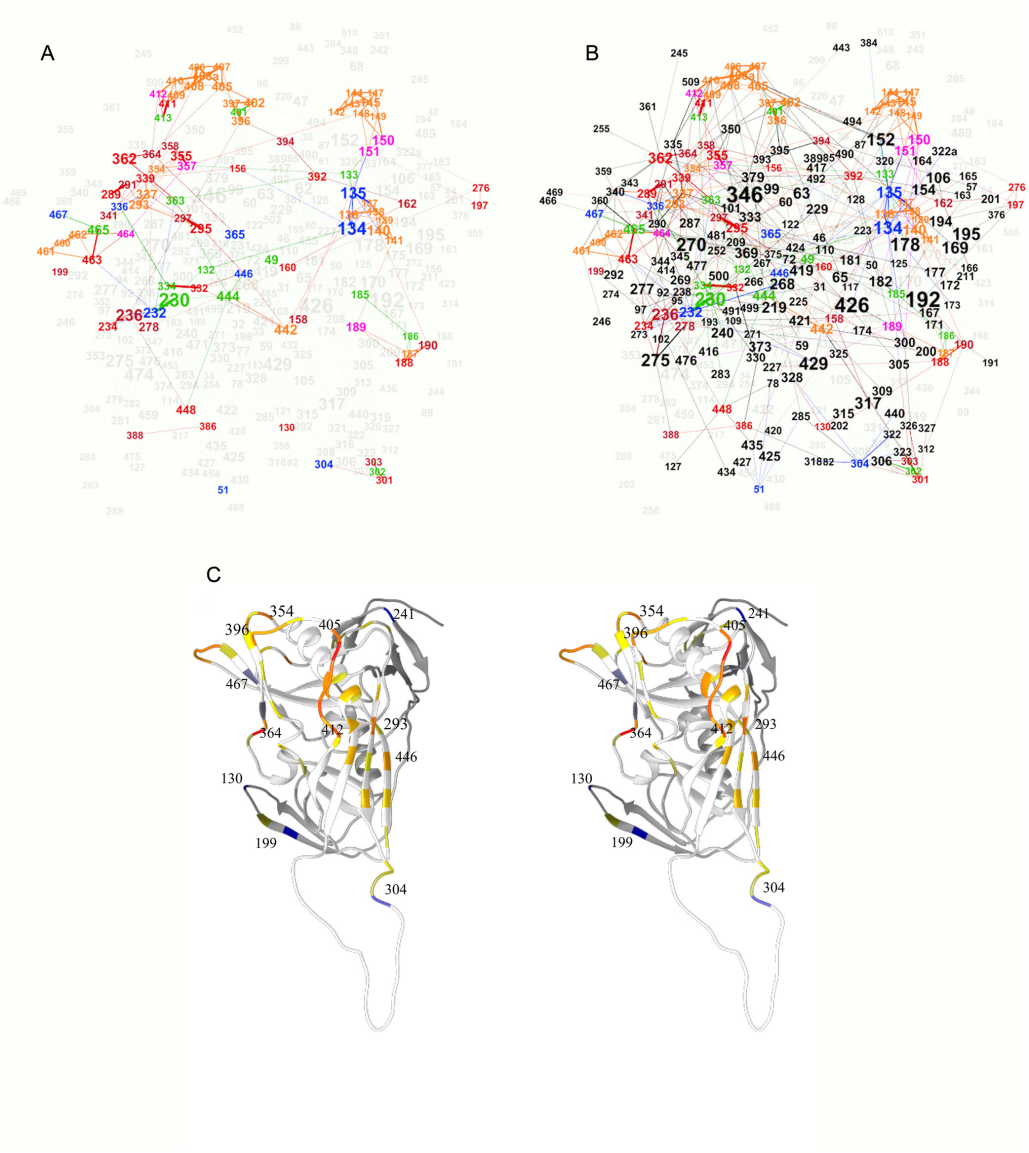
Mapping the sequon related positions of the potential N-glycosylation sites on the graph of mfDCA couplings. (A) “Variable”—ochre for N and magenta for S/T, “Frequent”—red –N, bordeaux –S/T and “Rare”—green—N, blue—S/T for. The P3 (S/T) positions appear less than the P1 (N) positions because N was selected to represent the position when the P1 and P3 overlapped for adjacent sequons. Overall, the sequons formed two connected subgraphs leaving out positions 49, 51, 130 and 199. The variable V1/V2, V4 and V5 regions represented tight cliques in the large subpgraph while p301–304 formed the smaller subgraph. The ego network (all immediately connected positions) of the sequons (B), was a highly connected and included a large part of the non-sequon positions. Therefore, it seems most of the sequons are closely interrelated but do not segregate from the rest of the structure. Even the variable loops cliques are bound by numerous relations to the rest of the graph. (C)–The sequon related positions mapped on the 2B4C structure in stereo view are color coded for their epistatic coupling to other sequon positions (red), to non-sequon positions (blue) or to both equally (yellow). If the proportion of sequon position related couplings is above 0.5 the color is orange, if less than 0.5 –gray. The predominantly sequon coupled positions are found in V4 but mostly in V1/V2 (not shown) as well as 293 and 446 in the middle of the cluster on the outer domain. The most “structure related” sequon positions are 130, 199, 304, 368, 467 and 241.

Mapping the sequon related positions on the coevolution graph reiterated their grouping in variable loop and “constant” part ones (Fig 5A). The SRP alone formed a rather connected network leaving out the V3 sequons (N301/ T303 and N302/R304) and the N386/T388 sequon while the inner domain positions T49, T51, N130 and S199 were not dependent on any other sequon. The sequon ego network (all positions immediately linked to a SRP) contained the larger part of the non-sequon positions (144 of 233 nodes–Fig 5B) and represented a highly connected network. Even the variable loop cliques are bound by numerous relations to the rest of the graph. The SRP that had relation to non-sequon positions were 85 of 94. Interestingly, 6 of 8 SRP that related only to other SRP were found in V1/V2 and the other two are S364 and N406 in V4. The sequon unaccounted for is the isolated node T415. The sequons that had predominantly non-sequon dependencies were found on the constant part in close vicinity to the loops (Fig 5C). These latter positions were also related to the most connected core of the co-evolutionary network.

### The central positions

**Fig 6:**
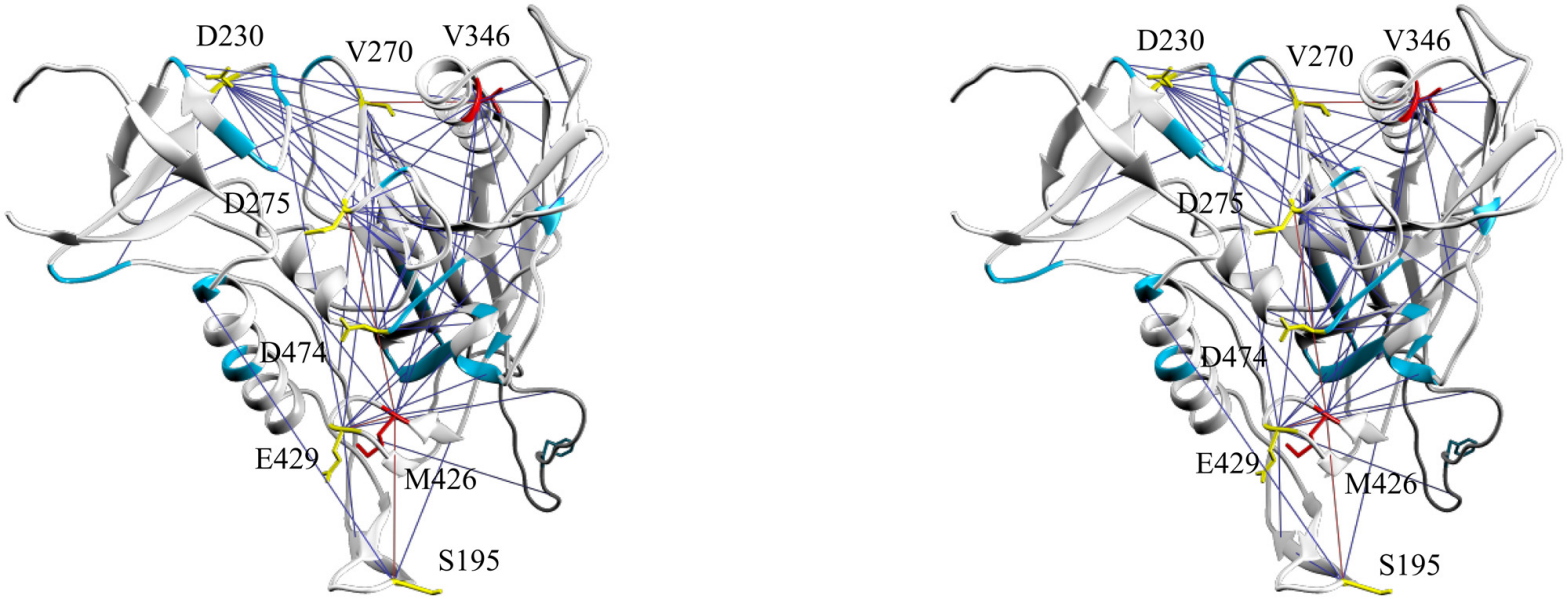
Map of the sites with the highest betweenness centrality values on the 2B4C structure—a stereo view. Red—group 1, yellow—group 2, cyan—group 3 (only positions in groups 1 and 2 are labeled). These positions are found in the membrane proximal part of the inner domain, the bridging sheet and the CD4 binding site. The pseudo-bonds indicate the immediate mfDCA couplings (ego network) of the positions in the 3 groups. (The Chimera file is S4 File).

The effect of the evolving glycan shield on the rest of the structure was studied also in the context of the potential allosteric pathways. The latter were predicted as the shortest paths in the co-evolutionary graph. Betweenness centrality (BC) measures the proportion of shortest paths that a node lies on. In this way, BC can be used to predict the frequency with which a position participates in allosteric paths. The highest BC values clustered in three groups (S1 Fig) and the corresponding sites (3, 9 and 29 sites respectively) represented a core structure highly connected to all other sites. Fig 6 (and S4 File) maps the 29 sites common with 2B4C color coded for the group. In descending order of the centrality measure associated they represent: group 1—V346, R192 and M426; group 2—**D230**, K178, V270, D474, **L134**, E429, G152, E275 and S195 and group 3—V169, F317, D62, **T236**, Q170, **N135**, T63, E268, A219, **N362**, E106, F277, I154, R419, V65, N99, V182, P369, E47, I194, **T140**, G471, **Q442**, G379, M373, **K232**, **R444**, **E150** and T240. None of these sites lied on paths that connected less than 149 other sites and 10 (underlined) were on shortest paths connecting more than 300 sites. The sites considered connected were those at the termini of the paths only and not the intermediates. Among the positions with the top 41 BC values there were 10 SRP (bolded), a proportion not significantly different from the overall. Neither did the distribution of SRP correlate with the classification in 3 groups with top BC. Thus, being a sequon related position did not correlate with allosteric centrality. Some SRP participated in the majority of the allosteric paths, e.g.—N362 (degree = 9) sits on allosteric paths that connect all nodes in the main component of the graph (n = 323), T236 (degree = 13) and N135 (degree = 12) each connect 297 nodes, etc. Other SRP, mostly those in the V4 and V5, were only locally connected. The node degree, the number of terminal positions of the paths involved and the BC values correlated only weakly. Interestingly, 12/41 top BC positions were found in V1/V2 region. It seems to have a central role in the structural dependencies despite its disordered conformation and much more so than the other variable regions. Group 1 and 2 of the top BC positions (n = 12) were found in the CD4 binding region, some membrane proximal positions (D230, V270 and D275), V3 (F317) and the α2-helix (V346—degree = 16, connects 289 sites, focuses most paths and is the most central position according to this graph). This set generally forms an intermediate region connecting allosteric paths that span the inner and the outer domains.

### Long range interactions and SRP

**Fig 7:**
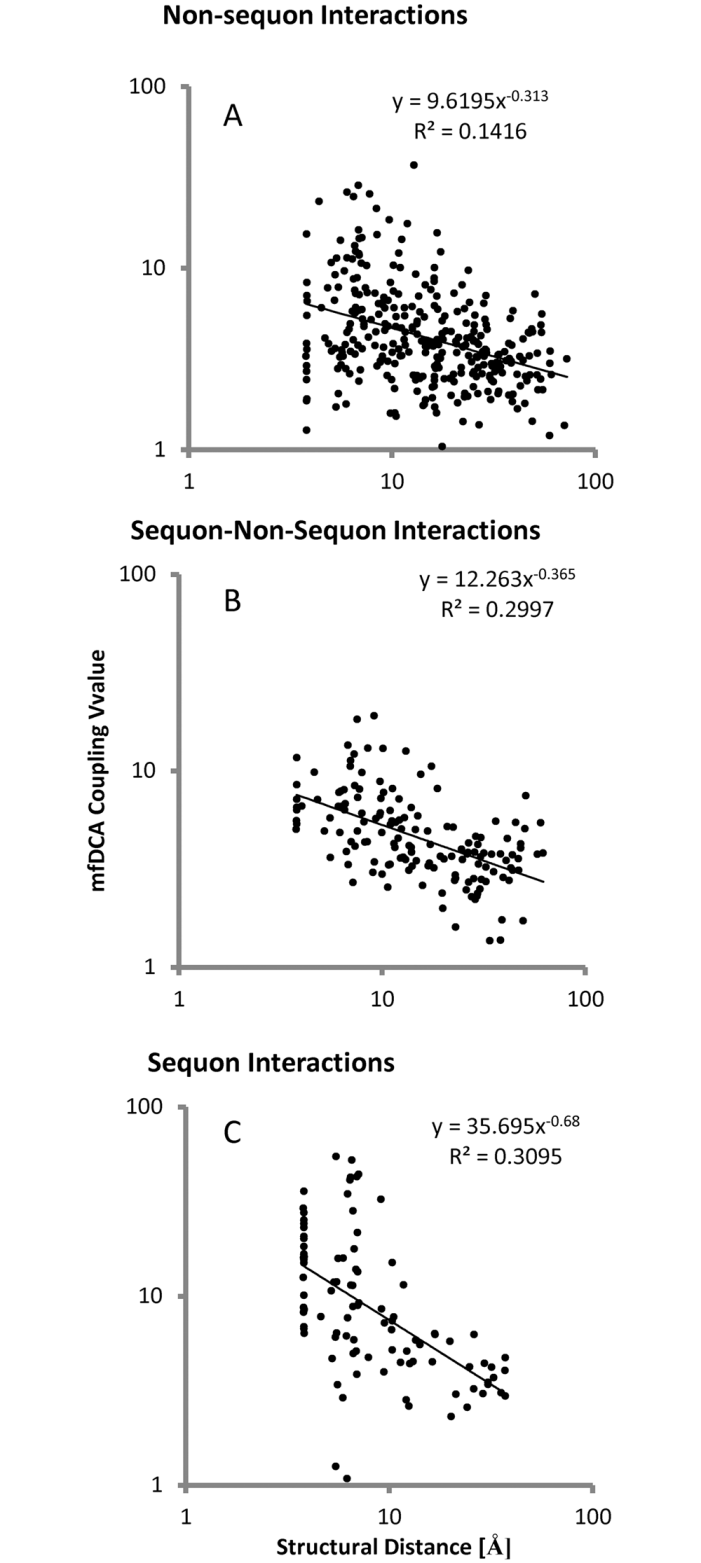
Dependence of mfDCA values on distance. As expected, the magnitude of the mfDCA coupling decayed with the increase of the distance between the pair of interacting positions. Both non-sequon related (A) and sequon to non-sequon related interactions (B) showed a rather regular pattern of interaction strength inversely proportional to the distance. Sequon to sequon interactions magnitude fell off with the distance faster (C) and were almost entirely contained within a radius of 15Å consistent with contact interactions between glycans. A small group of inter-sequon interactions occurred at a distance more than 20 Å.

**Fig 8:**
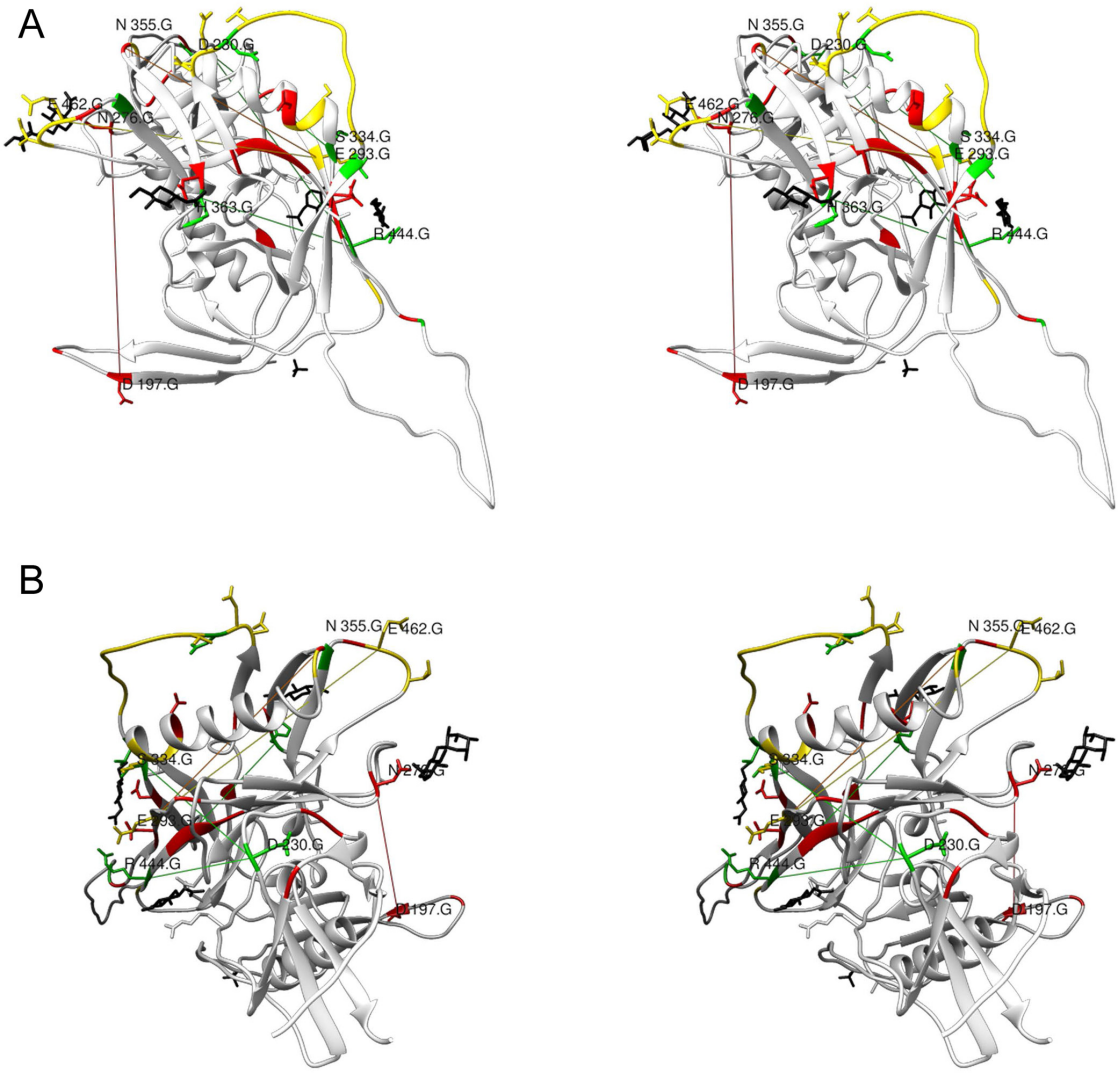
The long range sequon to sequon interactions mapped onto the 2B4C structure. A—Stereo view perpendicular to the axis of the trimer, B—stereo view form the membrane proximal region along the axis of the trimer. The sequence related positions are color coded for their frequency/entropy (rare—green, variable—yellow, frequent—red). For a more precise inspection see the Chimera file (S3 File).

**Fig 9:**
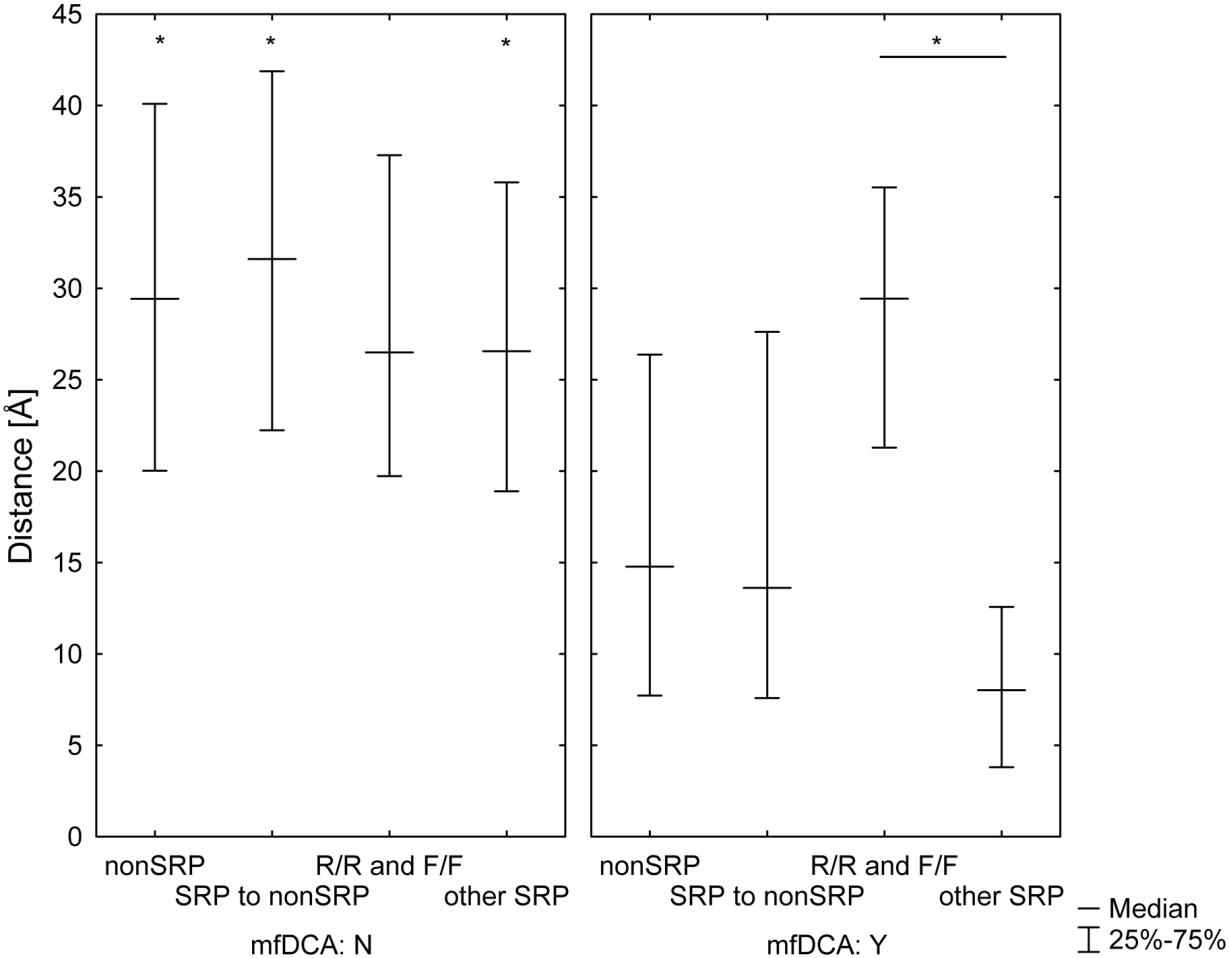
Difference in the pair distances of the couplings between sequon and non-sequon positions based on the structure 2B4C. The plot represents medians and interquartile ranges. The intersequon pairings (between positions 1 and 3 in each sequon) were excluded from the analysis. All pairs of positions were grouped first according to the significance of their mfDCA coupling: A not significantly correlated, B with significant mfDCA couplings. Then all positions were grouped in the following groups: non-sequon related—nonSRP, frequent sequon related positions—F, rare sequon related positions—R and variable sequon related positions—V The pairings were further grouped in those between nonSRP, between nonSRP and SRP, the homotypic pairings between F or R and finally—all other intersequon couplings. In this way, the typical distances for the interacting pairs were compared to the remaining pairs of the same type existing in the structure. The distances were compared using Kruskal-Wallis based multiple comparisons test (* - p<0.05). The mfDCA significant couplings were at shorter distances than the non-significant ones except for the homotypic pairs between frequent or rare sequons (F/F and R/R). The interacting homotypic pairs F/F and R/R were also at a longer distance than all other interacting SRP pairs Overall, these results indicate that homotypic interactions between frequent and rare SRP are at long distances and this is not because they are positioned distinctly in the structure since the mfDCA non-significant pairs among them are not different from the rest.

As expected, the mfDCA values decreased with the increase of the interatomic distances (Fig 7). SRP couplings decreased faster than the rest. The large radius of the glycans increases the distance, at which direct contacts would affect fitness Thus, it seemed that SRP coevolution was predominantly affected by direct contacts between amino acid residues or by the attached glycans. At the same time, several long range interactions were found too (Fig 8, S3 File). These included E293–N462, E293–N355, S334–D230, R444–D230, R444–Q363 and N197–N276. Most of them involved the outer domain SRP E293, S334 and R444 and linked them to membrane proximal regions (D230, N355 and N462), and the core near V4/V5 (Q363 and N355). Although these long range interactions were few, they represented homotypic pairing between low entropy positions (both frequent and rare) but not highly variable sequon positions (Fig 9 and Table 1). The long range homotypic frequent to frequent and rare to rare sequon pairings together with much closer frequent to rare interdependencies confirmed that rare sequons most probably are versions of the nearby frequent ones and probably have a similar role. Homotypic coupling is predominant also for variable loops high entropy SRP, which are mostly connected to their neighbors.

**Table 1:** Ratio of actual over expected frequencies (based on marginal frequencies) of interactions (mfDCA) between different sequon related positions. The interactions are distributed unevenly (X^2^, p<0.001). As expected there is a preference to N-S/T relationship obviating the epistatic link in the individual sequons. Further, variable sequons are related mostly to themselves forming cliques in the variable loops. The preference of frequent for rare N positions indicates that the rare sequons may be often variants of the frequent. At the same time S/T positions of variable sequons are related also to N positions of rare sequons.

	N of Frequent	N of Variable	N of Rare	S/T of Frequent	S/T of Variable	S/T of Rare	Non-Sequon
**N of Frequent**	0.89						
**N of Variable**	0.87	2.40					
**N of Rare**	1.38	0.90	0.32				
**S/T of Frequent**	2.00	1.11	1.04	0.28			
**S/T of Variable**	1.18	2.31	1.63	0.44	1.39		
**S/T of Rare**	0.65	1.06	2.24	0.73	1.15	0.21	
**Non-Sequon**	0.92	0.66	0.92	1.00	0.65	0.94	1.11

## Discussion

Here we attempted to give as complete a description of the co-evolutionary dependencies in HIV gp120 of Clade B as currently possible using the available sequences at the LANL and the mean field direct coupling analysis. A special emphasis is laid on the network of interactions of the potential N-glycosylation sites in the context of the immune aspects of the evolving glycan shield [8, 27]. A description of their functional relations was considered of theoretical and vaccinological interest. In particular, it was interesting to delineate the homotypic interactions between the sequons as shaped potentially by external pressure—mostly antibody neutralization, was expected, a considerable number of sequon/non-sequon correlations were established. What is more exciting—the set of SRP was found to be immediately linked to 62% of the studied positions indicating that even if the immune pressure shapes the evolving glycan shield, the consequences affect the entire structure.

Classifying the SRP according to their conservation demonstrated that the most variable group (in the variable loops) as well as the more conserved ones both show abundance of homotypic SRP to SRP interactions. In the case of the SRP that are found on the core, these homotypic interactions were predominantly at a long distance. One explanation for these findings could be the ultimate necessity to preserve the glycan shield in the evolutionary process leading to replacement of lost sequons. A leading cause for this preservation should be related to its antibody shielding function. On the other hand, long range direct dependencies cannot be explained by immune pressure easily if shielding of the adjacent protein core epitopes is at stake. Alternatively, they can be due to electrostatic interactions– or to interaction between the protomers in the homotrimer. So far, only the interaction of the V1/V2 loop from the neighboring protomer has been demonstrated indirectly [28]. It is possible that the neighboring V1/V2 interacts not only with V3 but also with the adjacent area of the outer domain including the residues that appear highly connected in beta13 and alpha2. It is not always easy to distinguish inter- from intraprotomer masking with respect to V1/V2 and V3 as Liu et al. showed in an elegant experiment [29]. Gycan interactions, on the other hand, can occur at much larger distance than amino acid side chains because of their mobility and size. Previously Poon et al. described evolutionary interactions between N-glycosylation sites [18]. The authors concentrated exclusively on the potential N-glycosylation sites based on an alignment of 711 sequences from different clades. The covarion model was used to evaluate variation over time in the site-specific rates that potential N-glycosylation sites were gained or lost. The authors concluded that nearby sequons were linked by exclusive interactions (only one of the sites can contain sequon) while distant interactions were positive (sequons co-occur). Comparing the mfDCA graph to the results from Poon et al. shows that the results are in the same vein (Table 1). A number of interactions found by the covarion method appear in the mfDCA graph often including one or more intermediates, e.g.– T465-(V360)-N362, N295-(S446)-Q442, Q442-(R444)-D230, etc. Apart from the missing general co-evolutionary context in the Poon et al. data, the differences may be due also to the different sets of data as the authors looked at a more general picture of HIV evolution including fewer sequences from all clades.

The statistical nature of the direct coupling scores necessitates caution and confirmation in the interpretation of the functional bonds discovered. The overall agreement of our findings with the structural data for Clade B gp120 as well as the previous successful application of mfDCA for prediction of tertiary structure and contact residues across many protein families [6, 17] argue in favor of the conclusions at a more general level. Several attempts were made to describe co-evolutionary patterns of gp120 in the recent years. Early on Korber et al. [30] studied the covariance of mutations in the V3 loop of the envelop protein of HIV type 1. A group of connected positions including S306, H308, A316, F 317, G321 and E322 was identified. A good agreement was found between this data and the mfDCA network, which showed a highly connected clique containing these and adjacent V3 residues. Korber at al. did not correct for the entropy and chain effects but instead calculated statistical significance only by a bootstrap procedure. It is possible that the clique type of connectivity allowed for an equivalence between the two methods. Wei et al. analyzed separately B and C clade sequences and found considerable differences in the C terminal part, which evolves much faster in clade C [10]. The technique used to filter the noise tends to lose information resulting in unexpectedly low number of co-evolving sites. Sethi et al. looked for allosteric coupling using correlated movement in the gp120 structure subject to molecular dynamics simulation [31]. The emphasis of this approach was the allostery, which is one of the possible sources of selection pressure resulting in epistasis. The communities, the authors defined, partially correspond to the modularity picture presented in this report with the obvious consensus on variable loops but with less agreement in the core of the gp120. The lack of V3 in the structure Sethi et al. used makes the comparison even more difficult. Perhaps the most interesting parallel is the prediction of one particular allosteric path. While the one end was chosen to represent a key residue in the CD4 binding cite—position F353, the other end of the path in the C-terminal part of the α2 helix—P369, seems chosen somewhat arbitrarily. The authors found a path of 5 positions (E466, V360, N362, S364 and V372) that linked these two points. In the mfDCA graph the shortest path between the two positions is represented by a single position—F277. There are also alternative longer paths in the mfDCA graph that include the listed positions. An important factor that may underlie the discrepancies is the highly truncated structure used for the molecular dynamics simulations. It lacked glycans and variable loops. Interestingly, in the allosteric path F353, E466, V360, N362, S364, V372 and P369 the underlined positions represent a sequon. In the path F353, F277, P369 found by mfDCA none is SRP.

The glycan shielding of the gp120 from antibodies is not absolutely effective. For instance, antibodies that bind epitopes on the glycans can be elicited. Although the intrinsic carbohydrate determinants are “self”, their arrangements in higher order structures (from adjacent glycans) are identified as “non-self”. Thus, the evolutionary pressure does not come only as a necessity for shielding the protein core but also to shuffle potentially epitopic higher order structures [32, 33]. A number of studies exist describing in detail evolutionary events that affect gp120 sequons [34–36]. Together they describe a supersite of vulnerability through glycans, which participate in most of the broadly neutralizing anti-carbohydrate antibodies epitopes. The prototype antibody 2G12 binds to glycans attached to (N295, N332, N339, N386, and N392) [37], the antibody PGT135 has an epitope involving SRP N332, N386 and N392 [15] and PGT128—N332 and N301 [38]. Analysis of the neutralizing capacity of lectins and the resulting viral adaptations showed again a central role for positions N295, N332, N339 and N392 [35, 38, 39]. Thus, despite its unusual structure, 2G12 is not unique as specificity since other broadly neutralizing anti-carbohydrate antibodies and carbohydrate binding proteins bind to overlapping glycans and are similarly neutralizing. Dacheux et al. found that 2G12 epitope appears late in the evolution of the virus in individual patients, suggesting that 1) the antibody activity to this epitope is preexisting or appears early in the disease causing loss of the epitope and 2) the glycans involved are functionally important since the epitope is restored as soon as the immune function declines [27]. The mfDCA analysis is in agreement with this hypothesis. The SRP related to the sequons defining these N-glycosylation sites are of moderate centrality for the graph of interactions, but they are immediately linked to other positions of central role (participating potentially in a great many allosteric paths). For instance N332 is linked to Q442 (also SRP) and through it to the top BC value site E429 (Fig 6) and 334 is linked to another top BC value site D230, which is one of the most central positions according to the present analysis. Furthermore, sequons N339/T341 and N295/T297 are mutually linked but also depend on M426 and V270, which are two more top BC value sites. N295 itself is a top BC value site. It seems several allosteric paths from the α2 \V4\V5 region may be passing through N339 and N295. This puts constraints on these two sequons and can explain the tendency for reverting to sequences which contain them.

Thus, a comprehensive map of the putative co-evolutionary dependencies in Clade B gp120 made obvious the strong interactions between sequon related positions, which is partially explained by immune pressure on the evolving glycan shield. At the same time it mapped these interactions to the overall network of functional dependencies showing the major effect any evolutionary pressure on the glycan composition has on the protein core. These results, together with previous in vitro data by Balzarini [36] and Witvrouw [35], provide a basis for more efficient inhibitor strategies that force the virus in predetermined paths of adaptation.

## Supporting Information

S1 Dataset MSA, sequon list, significant mfDCA and Betweenness Centrality values as Excel spreadsheets. (XLSX)

S2 Dataset MSA used as a text file. This file is referred to as R.txt in S2 File.
(TXT)

S1 Fig Positions ranked by betweenness centrality values. (PDF)

S2 Fig mfDCA vs distances between Calpha atoms in 2B4C structure. (PDF)

S1 File A text file with the description of the graph of couplings in vna format. (TXT)

S2 File The Matlab script used for scrambling the columns of MSA. (TXT)

S3 File Chimera (Python) file of 2B4C structure with long range inter sequon relations as pseudobonds. (ZIP)

S4 File Chimera (Python) file of the 2B4C structure with top betweenness centrality positions labeled and their couplings shown as pseudobonds. (ZIP)
