# Supplementary figures and images for: The Patterns of Coevolution in Clade B HIV Envelope's N-Glycosylation Sites
Source: PLoS One. 2015 Jun 25;10(6):e0128664. doi: 10.1371/journal.pone.0128664 (PMC4482261; doi:10.1371/journal.pone.0128664)

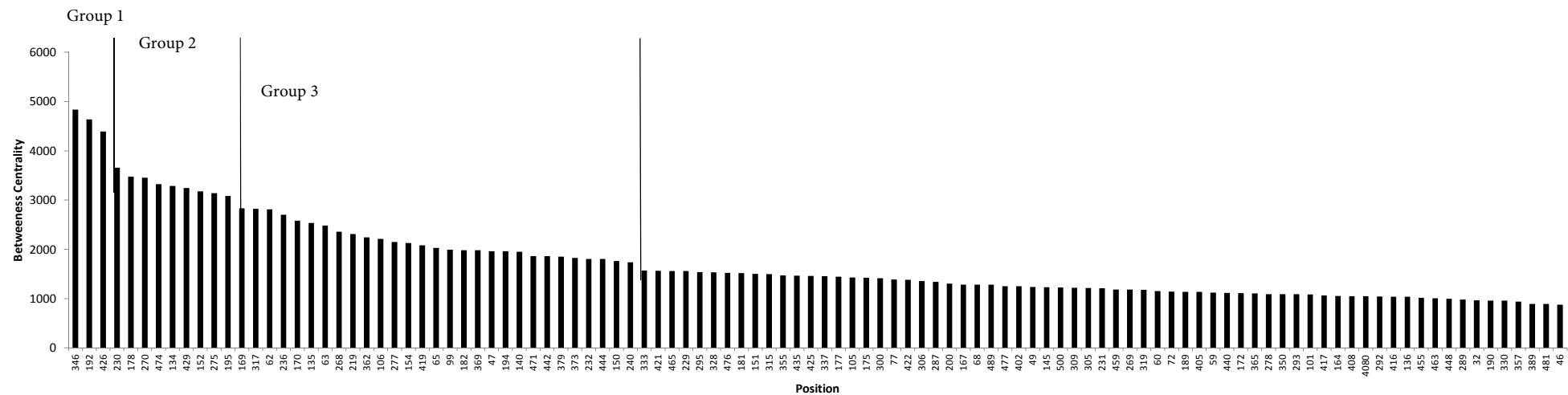

Supplement: S1 Fig — (PDF) [file pone.0128664.s003.pdf]
