# Supplementary material for: The Patterns of Coevolution in Clade B HIV Envelope's N-Glycosylation Sites
Source: PLoS One. 2015 Jun 25;10(6):e0128664. doi: 10.1371/journal.pone.0128664 (PMC4482261; doi:10.1371/journal.pone.0128664)
Supplement: S2 Fig — (PDF) [file pone.0128664.s004.pdf]

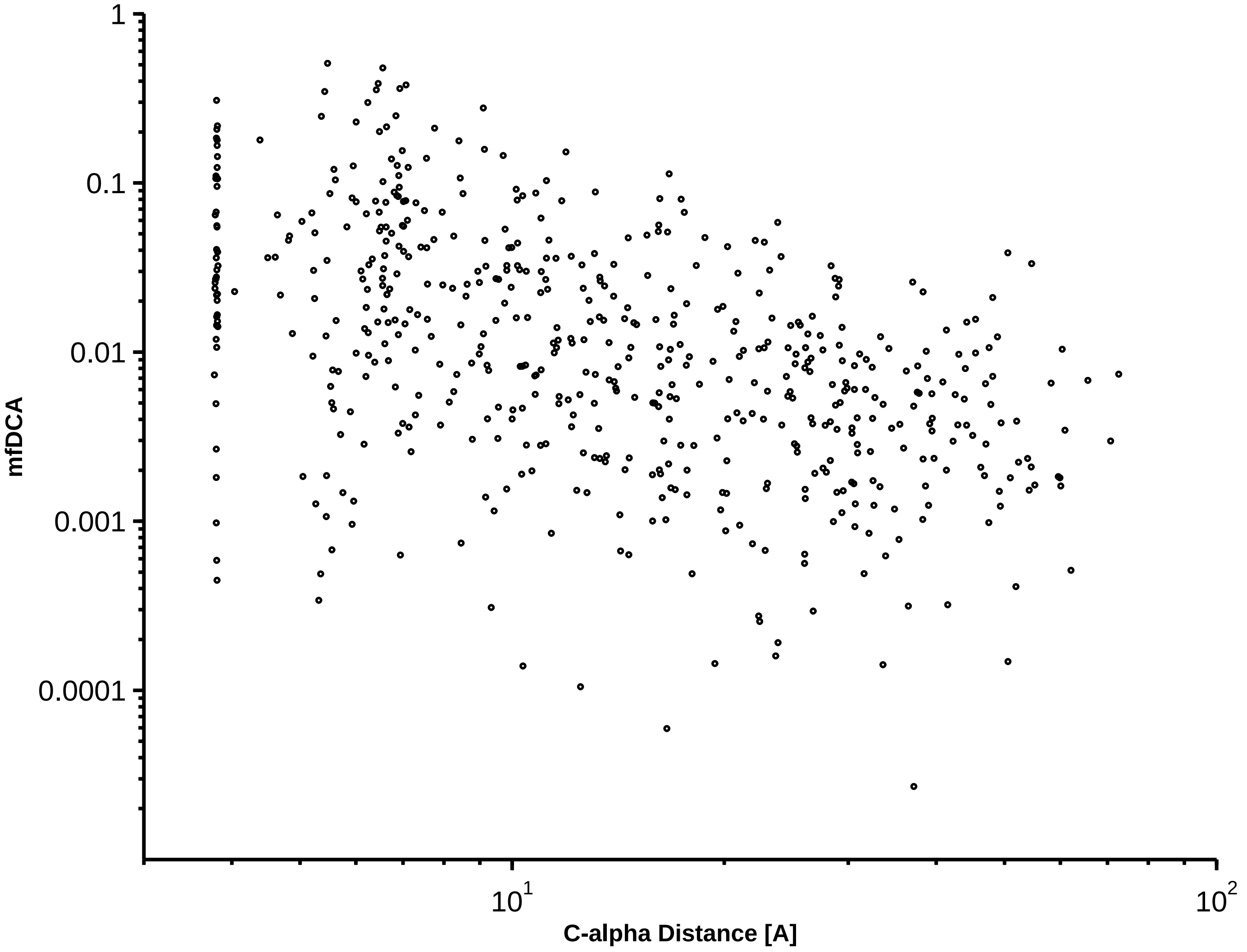

S8 Fig. As expected, the intensity of the mfDCA correlation for the significant couplings decreased with the distance and this decrease was roughly propotional to the square of the distance between the Cα atoms of the residues.
